# A Comparison of Outcome of Osteoarticular Allograft Reconstruction and Shoulder Arthrodesis Following Resection of Primary Tumours of the Proximal Humerus

**DOI:** 10.1080/13577149877920

**Published:** 1998-12

**Authors:** L. J. Probyn, J. S. Wunder, R. S. Bell, A. M. Griffin, A. M. Davis

**Affiliations:** 1 University Musculoskeletal Oncology Unit Mount Sinai Hospital Toronto Canada; 2 Department of Surgery University of Toronto Toronto Canada; 3 Departments of Physical Therapy and Surgery and Graduate Departments of Rehabilitation Science and Public Health Science University of Toronto Toronto Canada; 4 Suite 476 Mount Sinai Hospital 600 University Avenue Toronto M5G 1X5 Canada

## Abstract

*Purpose.* The purpose of this study was to compare the oncologic, reconstructive and functional outcomes of patients who underwent osteoarticular allograft reconstruction with those who underwent arthrodesis for a primary malignant bone tumour of the proximal humerus.

*Patients.* Eleven patients were treated with osteoarticular allograft reconstruction of the proximal humerus. Five of these reconstructions failed, necessitating revision to a secondary arthrodesis. Five patients underwent arthrodesis as a primary reconstruction, for a total of ten patients in the arthrodesis group.

*Methods.* End points included local and systemic disease recurrence, complications and functional outcome (measured using the 1987 and 1993 Musculoskeletal Tumour Society Rating Scales (MSTS) and the Toronto Extremity Salvage Score (TESS)).

*Results.* One patient died of systemic disease 2 years post-operatively and one patient had an axillary node recurrence resected 10 months post-operatively and remains free of disease 53 months later. The other 14 patients were alive with no evidence of disease at the time of the last follow-up. Complications after the osteochondral allografts (*n*=11) included two infections, four fractures and three subluxations in eight patients. Six of these patients required removal of the allograft; one had a repeat osteochondral allograft and five were converted to an arthrodesis. Complications after arthrodesis in the ten patients (five primary and five secondary arthrodeses) included two non-unions, one infection and one fracture in three patients. Patients who underwent shoulder arthrodesis scored better in all outcome measures and this was statistically significant in the MSTS 1993 (*p*=0.001, Mann–Whitney U Test).

*Discussion.* In this study, there was a trend towards improved function following arthrodesis compared to osteochondral allograft reconstruction following proximal humerus bone tumour resection.


*Sarcoma* (1998) **2**, 163–170

ORIGINAL ARTICLE

### A comparison of outcome of osteoarticular allograft reconstruction and shoulder arthrodesis following resection of primary tumours of the proximal humerus

L. J. PROBYN,[1] J. S. WUNDER,[1,2] R. S. BELL,[1,2] A. M. GRIFFIN[1] & A. M. DAVIS[1,3]

[1]*University Musculoskeletal Oncology Unit, Mount Sinai Hospital &* [2]*Department of Surgery, University of Toronto &* [3]*Departments of Physical Therapy and Surgery and Graduate Departments of Rehabilitation Science and Public Health Science, University of Toronto, Toronto, Canada*

**Abstract**

*Purpose.* The purpose of this study was to compare the oncologic, reconstructive and functional outcomes of patients who underwent osteoarticular allograft reconstruction with those who underwent arthrodesis for a primary malignant bone tumour of the proximal humerus.

*Patients.* Eleven patients were treated with osteoarticular allograft reconstruction of the proximal humerus. Five of these reconstructions failed, necessitating revision to a secondary arthrodesis. Five patients underwent arthrodesis as a primary reconstruction, for a total of ten patients in the arthrodesis group.

*Methods.* End points included local and systemic disease recurrence, complications and functional outcome (measured using the 1987 and 1993 Musculoskeletal Tumour Society Rating Scales (MSTS) and the Toronto Extremity Salvage Score (TESS)).

*Results.* One patient died of systemic disease 2 years post-operatively and one patient had an axillary node recurrence resected 10 months post-operatively and remains free of disease 53 months later. The other 14 patients were alive with no evidence of disease at the time of the last follow-up. Complications after the osteochondral allografts ($n = 11$) included two infections, four fractures and three subluxations in eight patients. Six of these patients required removal of the allograft; one had a repeat osteochondral allograft and five were converted to an arthrodesis. Complications after arthrodesis in the ten patients (five primary and five secondary arthrodeses) included two non-unions, one infection and one fracture in three patients. Patients who underwent shoulder arthrodesis scored better in all outcome measures and this was statistically significant in the MSTS 1993 ($p = 0.001$, Mann–Whitney U Test).

*Discussion.* In this study, there was a trend towards improved function following arthrodesis compared to osteochondral allograft reconstruction following proximal humerus bone tumour resection.

**Key words**: *sarcoma, proximal humerus, reconstruction, outcome, function.*

### Introduction

Reconstruction of the shoulder following resection of a primary tumour of the proximal humerus is a challenging clinical problem, particularly since the resection may result in deficits of the deltoid, rotator cuff, joint capsule, glenoid and scapula. The reconstructive options following proximal humerus tumour resection include: preservation of a mobile glenohumeral joint using a prosthesis, osteoarticular allograft or an allograft–prosthesis composite; arthrodesis using an allograft or fibular transplant; or, a flail joint (Tikhoff–Linberg procedure).[1–4] When sufficient deltoid, rotator cuff and joint capsule can be preserved to power the glenohumeral joint, our group has undertaken reconstruction with osteoarticular allograft. When insufficient soft tissues remain, we have utilized an allograft arthrodesis. When resection of the proximal humerus, glenoid and scapula leaves insufficient bone stock to permit shoulder fusion, the Tikhoff–Linberg procedure is undertaken to avoid forequarter amputation whenever possible.[5,6]

The perceived advantage of an osteoarticular allograft reconstruction compared to shoulder fusion is based on the potential for improved function due to a mobile glenohumeral joint. However, there is often considerable mobility imparted to the upper extremity by the scapulothoracic joint following arthrodesis. There is no information in the literature comparing the results of a mobile shoulder versus

Correspondence to: A. M. Davis, Suite 476, Mount Sinai Hospital, 600 University Avenue, Toronto, Canada M5G 1X5. Tel: + 1 (416) 586-8678; Fax: + 1 (416) 586-8397; E-mail: davis@mshri.on.ca.

1357-714X/98/030163-08 $9.00 © 1998 Carfax Publishing Ltd arthrodesis reconstruction following proximal humerus tumour resection. The purpose of this study was to compare the oncologic, reconstructive and functional outcomes of patients who underwent osteoarticular allograft reconstruction with those who underwent arthrodesis for a primary bone tumour of the proximal humerus.

### Methods

Patients were eligible for this study if they had resection of a primary bone tumour of the proximal humerus and reconstruction with an osteoarticular allograft or shoulder arthrodesis. All patients had a minimum of two years' follow-up from their index surgery.

Between 1986 and 1995, eleven patients were identified who underwent osteoarticular allograft reconstruction of the proximal humerus. During the same period, ten patients underwent shoulder arthrodesis. Five of these ten were initially treated with an osteoarticular allograft that subsequently failed. These five patients underwent arthrodesis as a secondary procedure.

All patients underwent clinical, radiographical (computerized tomography in the initial years and then both computerized tomography and magnetic resonance imaging in the latter years), and pathological staging prior to surgery.

All resections were classified according to the system of the Musculoskeletal Tumour Society.[7] If the abductor mechanism (deltoid and rotator cuff) was intact following tumour resection, the case was denoted with an A, or a B if the abductor mechanism was disrupted. All of the osteoarticular allografts were classified as S345A. At least a portion of the deltoid was disrupted in all of these cases but the rotator cuff remained intact. All of the primary arthrodeses (that is, arthrodeses done immediately following tumour resection, rather than to salvage a failed osteochondral allograft reconstruction) were classified as S345B and had resection of their rotator cuff and joint capsule as well as the deltoid.

### Reconstructive procedures

Tumour resection was performed following accepted oncologic principles aiming for negative operative margins. The status of the abductor mechanism was the most important factor in selecting the reconstruction method.[4] An osteoarticular allograft was performed if the rotator cuff and capsule (and rarely the deltoid) were intact, and an arthrodesis was performed if the rotator cuff, capsule and/or glenoid surface were resected (Figs 1 and 2). If there was involvement of the scapula including the glenoid with or without the proximal humerus, a Tikhoff–Linberg procedure was performed providing the neurovascular structures could be preserved. The patients undergoing Tikhoff–Linberg procedures were not included in this study.

### *Osteoarticular allograft*

An osteoarticular allograft of appropriate size (size match performed using standardized radiographs) was chosen from the bone bank prior to surgery. All allografts were harvested and stored according to the standards of the American Association of Tissue Bank[8] and all grafts were treated with 2.5 megarads of radiation following harvest. Osteoarticular allografts were thawed in the operating room and cut to fit the humeral defect. Prior to fixing the allograft to host humerus, a rotator cuff repair was performed

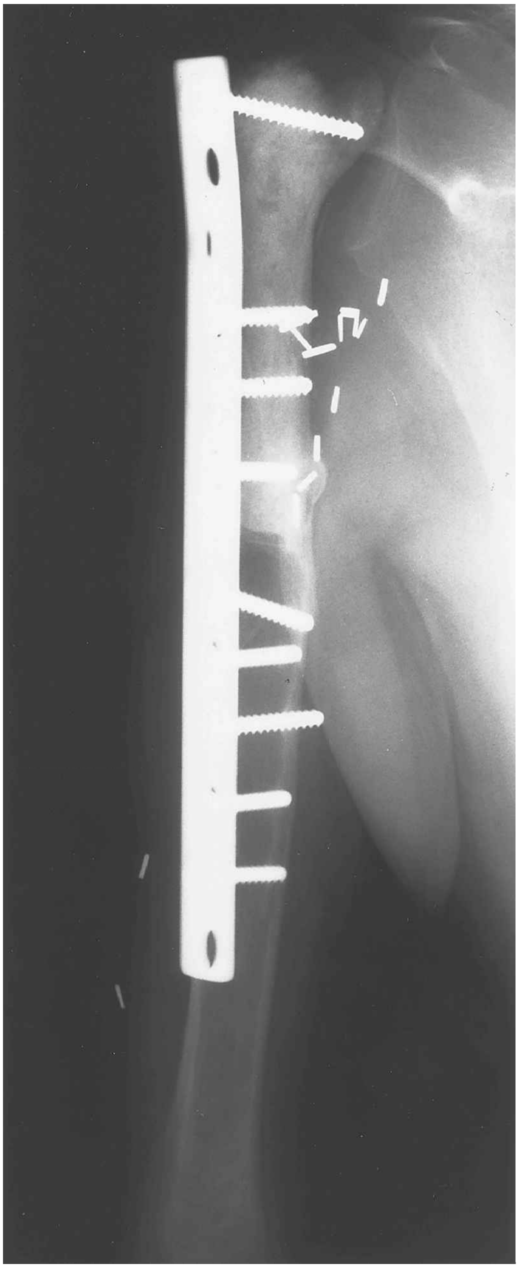

**Fig. 1.** *Anteroposterior plain radiograph of a 35-year-old man 4.5 years post-osteochondral allograft of the shoulder for a grade 2 chondrosarcoma.*

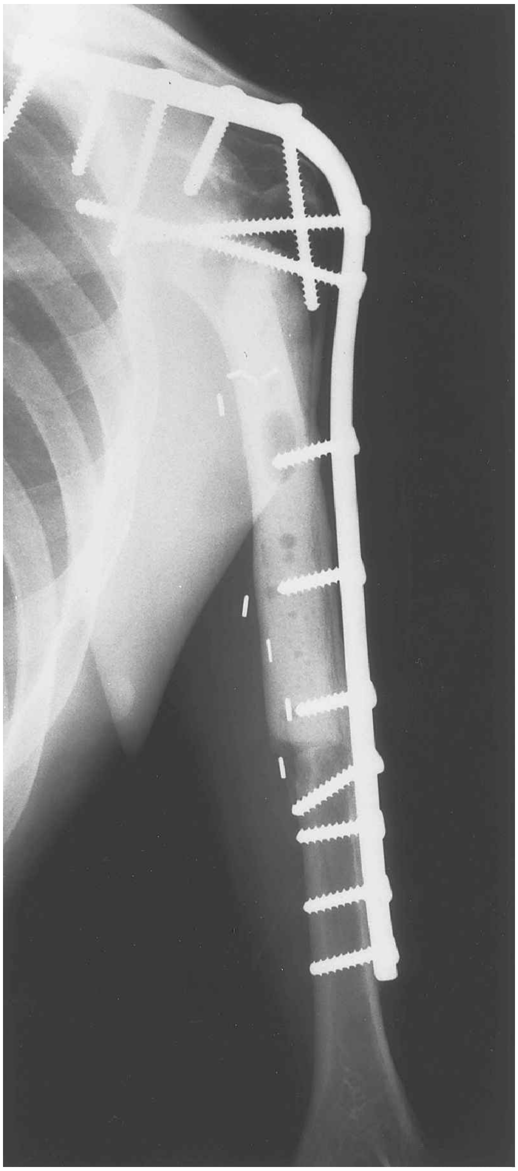

**Fig. 2.** *Plain radiograph 2 years following allograft arthrodesis for a grade 1 chondrosarcoma in a 19-year-old woman.*

between the host and the allograft tendons using non-absorbable sutures. A stable repair of the rotator cuff was achieved in all cases. Fixation of the allograft to host humerus was then performed using a dynamic compression plating technique. Autograft from the iliac crest was placed around the osteotomy site in eight of the eleven cases. The insertions of the latissimus dorsi and pectoralis major muscles were repaired to the corresponding areas on the allograft if possible. The biceps, brachialis and triceps were advanced for soft tissue coverage of the allograft.

### *Arthrodesis*

Eight of the eleven arthrodeses performed in ten patients were achieved using allograft. The allograft was prepared by osteotomizing the articular surface so that the remaining humeral head was congruent with the glenoid and acromion at thirty degrees flexion, thirty degrees of abduction and thirty degrees of internal rotation.[9,10] Soft tissues and cartilage were cleaned from the under-surface of the acromion. A very long (18–26 hole) 4.5 mm broad plate was contoured to lie along the spine of the scapula, over the acromion and down along the allograft onto the patient's remaining distal humerus. Interfragmentary compression screws were used following provisional stabilization with K-wires and passed through the plate and allograft, obtaining purchase into the glenoid and neck of scapula. Further interfragmentary screws were then passed through the acromion into the graft to achieve firm contact with the superior portion of the allograft humeral head. A dynamic compression plating technique was then used to stabilize the distal osteotomy between the allograft and the host humerus. Neutralization of the reconstruction was accomplished by insertion of screws through the plate into the allograft and the scapular spine. Iliac crest bone graft was packed around the proximal and distal osteotomies.

Three arthrodeses were performed using a vascularized fibular graft instead of an allograft as each of these had developed an infection; two following an osteoarticular allograft (Fig. 3) and one following an allograft arthrodesis. The infections were treated in a two-stage process with removal of the allograft and replacement with a cement spacer until the infection cleared and then subsequent arthrodesis with the vascularized fibular graft. A broad plate was contoured along the spine of the scapula to the distal humerus. A microvascular fibula was harvested and a hole was reamed in the glenoid where the fibular graft was slotted into place. The distal humerus was also reamed and the fibula was inserted about 2–3 cm into the humerus. The fibula was held in place proximally by a single screw through the fibula into the glenoid. The plate was secured to the fibula with screws. The microvascular repair was then performed with an end-to-side repair between the brachial artery and vein and the peroneal artery and vein. Iliac crest bone graft was applied at both anastomosis sites.

A latissimus dorsi muscle flap was required in five of the eleven arthrodesis reconstructions to provide complete soft tissue coverage over the graft site.

All patients in the osteoarticular and arthrodesis groups received peri-operative antibiotics and were immobilized in a Velpeau sling for 6 weeks. The osteoarticular allograft patients were started on physiotherapy at 6 weeks post-surgery with shoulder shrugs and active-assisted forward flexion exercises. Their rehabilitation programs were progressed as tolerated and according to radiographic evidence of healing. The arthrodesis patients were started on range of motion (ROM) exercises of the scapulothoracic joint when there was radiographic evidence of healing of the glenoid osteotomy. All patients were

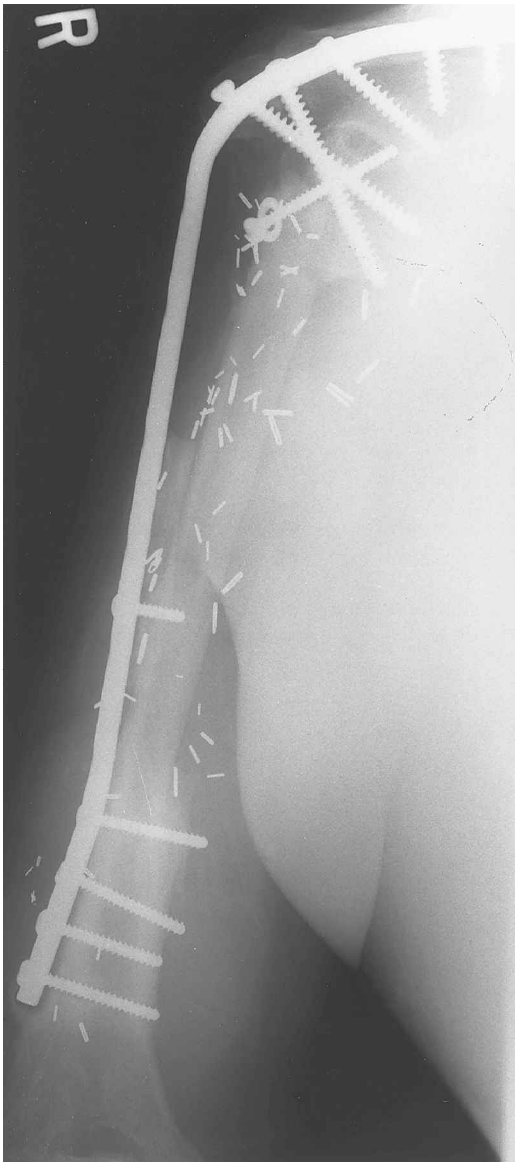

**Fig. 3.** *Plain radiograph of a vascularized fibular graft arthrodesis 3 years after surgery to revise an infected osteoarticular allograft.*

started on early gentle ROM exercises of the elbow, wrist and hand.

Demographic, oncological outcome, treatment complication and functional data at most recent follow-up were collected for all patients. Function was recorded using the Musculoskeletal Tumour Society Rating Scale, 1987 version (MSTS, 1987),[11] the Musculoskeletal Tumour Society Rating Scale, 1993 version (MSTS, 1993),[12] and the Toronto Extremity Salvage Score (TESS).[13] The functional data of the six patients, who had an osteoarticular allograft (without revision to an arthrodesis), were compared to the 10 patients who had an arthrodesis using non-parametric statistics.

The five patients who had a primary osteoarticular allograft and went on to a secondary arthrodesis were interviewed and asked specific questions comparing the two different types of surgery with respect to function (activities of daily living, ADLs, leisure activities, work, and pain levels.

### Results

### *Oncologic results*

There were seven males and four females who underwent an osteochondral allograft, and their average age at surgery was 34 years (range, 10–78 years). Seven of the patients had osteosarcoma (two IB parosteal and five IIB central osteosarcomas) and four of the patients had chondrosarcoma (all IIB lesions).[14] Three males and seven females underwent an arthrodesis (five of the ten after failed osteoarticular allograft reconstruction). The average age at surgery was 22 years (range, 12–35 years). Of the five patients undergoing primary arthrodesis immediately after tumour removal, three had a IIB osteosarcoma, one a IB malignant giant cell tumour and one a IB chondrosarcoma. Table 1 summarizes the results.

The average length of bone resection was similar for the osteoarticular allograft and arthrodesis groups, 15.2 cm (range, 7.3–25 cm) and 17.3 cm (range, 7.5–25 cm) respectively. Adjuvant treatment varied depending on the grade and stage of the tumour. For the osteochondral allografts, six of eleven patients received chemotherapy. For the primary arthrodesis group, three of five patients received chemotherapy. None of the patients in the study was treated with radiotherapy. Nine of the patients undergoing osteoarticular allografts had negative margins and two patients had positive microscopic margins. Four of the five patients undergoing primary arthrodesis had negative margins and one patient had positive microscopic margins.

At the time of the last follow-up, all of the patients who had an osteochondral allograft were alive with no evidence of disease. One patient with osteosarcoma treated with an osteoarticular allograft (negative margin surgery at initial operation) developed a high axillary nodal recurrence ten months after initial resection. Fifty-three months after wide resection of this nodal recurrence, the patient remains disease-free. One osteosarcoma patient treated by arthrodesis developed lung and pelvic metastases eight months post-operatively and died of disease at two years. This patient had positive resection margins at the time of surgery but did not develop a local recurrence. The other patients reconstructed by arthrodesis were alive with no evidence of disease.

### *Reconstructive results*

Of the eleven patients who had an osteoarticular allograft, five patients required revision, all to a glenohumeral arthrodesis. Two of these patients sustained late infections (greater than six months

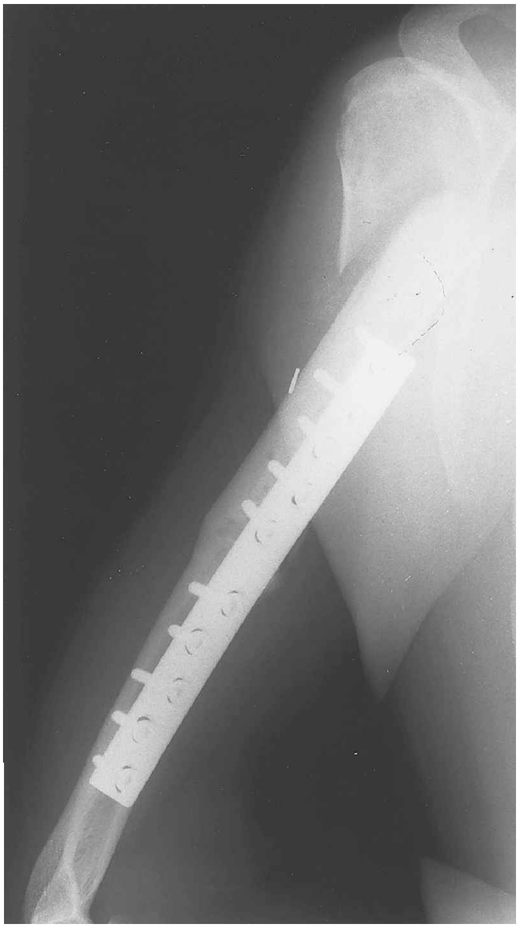

**Fig. 4.** *Plain radiograph showing an atraumatic fracture of an osteoarticular allograft 6 years post-operatively.*

post-surgery) and underwent staged salvage procedures with removal of the osteochondral graft, implantation of an antibiotic cement spacer and subsequent reconstruction using a vascularized fibula. Two patients with painful subluxation of the shoulder and one patient with an osteoarticular allograft fracture were also revised to an allograft arthrodesis. The length of time between the osteochondral allograft and the arthrodesis varied from 12 to 81 months (average 48.6 months).

Of the remaining six patients with osteochondral allografts, one patient developed an inferior dislocation of the allograft four months post-operatively. This was stable and the patient did not want further treatment despite limited glenohumeral motion. One patient sustained a fracture of the allograft after a fall and required replacement of the graft while two patients developed a fracture of the allograft without trauma. In one of these two patients, atraumatic fracture resulted in formation of callus around the allograft fracture site with eventual stabilization of the fracture, while the second patient is presently awaiting revision. Two patients have not had specific complications although one of these patients has moderate to severe shoulder pain but does not wish to undergo further surgery.

Of the five patients who had a primary arthrodesis, four patients have had no complications. One patient had a fracture of the allograft and developed chronic infection which was treated with a cement spacer and eventual microvascular fibular graft arthrodesis. Of the five patients who had a secondary arthrodesis, three patients have had no complications. Two patients sustained non-unions which healed after repeat bone grafting.

Functional data were available for all but one patient with an osteoarticular allograft who did not return for follow-up assessment. Mean follow-up time was 45.6 months (range, 24–81 months) for the osteochondral allografts and 48.6 months (range, 24–132 months) for the arthrodesis patients. Patients with a shoulder arthrodesis scored better in all outcome measures. The mean scores for osteochondral allografts and arthrodeses, respectively, for the MSTS 1987 was 19.3 (range, 7–27) and 21.1 (range, 17–25), for the MSTS 1993 was 50 (range, 36–70) and 68.2 (range, 53–80) and for the TESS score was 74 (range, 39–95) and 78.5 (range, 42–98). This difference was statistically significant only in the MSTS 1993 ($p = 0.001$, Mann–Whitney U) favouring improved function in the arthrodesis group. The active range of motion achieved by the two groups was especially striking. For all but one patient who experienced chronic, debilitating pain from an unstable osteoarticular reconstruction and continued to experience some pain following conversion to an arthrodesis, patients with an arthrodesis had better active forward flexion (range 45–85) than any patient with an osteoarticular allograft (range 30–70). This was reflected in the MSTS 1987 range of motion ratings in which no patient with an osteoarticular allograft received more than one of five points whereas eight of the ten patients in the arthrodesis group (five primary and five secondary arthrodeses) received three of five points for range of motion.

The subjective description of the comparison of the osteochondral allograft versus the arthrodesis from the five patients undergoing both surgeries seemed to support the trend favouring arthrodesis (see Table 2). Increased stability was noted after the fusion. The pain was also reported as the same or better after arthrodesis.

### Discussion

It has been well documented that limb-salvage surgery for tumours of the shoulder is an alternative to amputation.[4] Careful performance of the biopsy, the use of neoadjuvant chemotherapy when indicated and proper attention to complete surgical resection of the tumour are essential in permitting limb salvage without compromising tumour control. Successful local resection, avoiding amputation while controlling sarcoma, is demonstrated by the

**Table 1**. *Sample characteristics and outcomes*

| | *Osteochondral allograft** $n = 11$ | *Arthrodesis* $n = 5$ primary $n = 5$ secondary |
|---|---|---|
| Age (years) | mean = 33.6, sd = 21.5 | mean = 22.4, sd = 8.5 |
| Gender | | |
| Male | 7 | 3 |
| Female | 4 | 7 |
| Pathology | | |
| Osteosarcoma | 7 | 7 |
| Chondrosarcoma | 4 | 2 |
| Giant cell tumour | 0 | 1 |
| Adjuvant Rx | | |
| None | 5 | 6 |
| Pre-op chemo | 1 | 0 |
| Post-op chemo | 3 | 0 |
| Pre & Post-op chemo | 2 | 4 |
| Margins | | |
| Negative | 9 | 9 |
| Positive | 2 | 1 |
| Bone resection (cm) | mean = 15.2, sd = 4.5 | mean = 17.3, sd = 5.1 |
| Complications | | |
| Non-union | 0 | 2 |
| Infection | 2 | 1 |
| Fracture | 4 | 1 |
| Subluxation/instability | 3 | 0 |
| Functional outcome | | |
| MSTS 1987 | mean = 19.3, sd = 6.1 | mean = 21.1, sd = 2.4 |
| MSTS 1993 | mean = 50.0,sd = 9.8 | mean = 68.2, sd = 7.7 |
| TESS | mean = 74.0, sd = 23.5 | mean = 78.5, sd = 19.2 |
| Follow-up | mean = 45.6,sd = 20.6 | mean = 48.6, sd = 39.0 |

*Five patients with osteoarticular allografts were converted to arthodesis due to complications.

fact that there was only one nodal recurrence and one systemic relapse in the patients in this study. However, the choice of the best limb-reconstruction method for tumours of the proximal humerus is a difficult clinical decision.

In this study, patients were selected for osteoarticular allograft reconstruction when sufficient abductor musculotendinous tissue remained to provide a stable soft tissue repair to the rotator cuff and tendon of the allograft. Fusion was undertaken when the osteoarticular allograft failed due to infection, instability or fracture, or if the glenoid bone stock or soft tissues after resection were insufficient to reconstruct a stable, mobile shoulder. In both of these types of reconstructions, complications were frequent and serious. In the osteoarticular group, five have been revised to an arthrodesis: two for infection, two for instability and one for fracture. In the remaining six patients, three further fractures were documented (one requiring allograft replacement and one for which revision surgery is still pending), one patient dislocated the shoulder and one patient has chronic pain of moderate intensity. In comparison, three of the ten arthrodeses were complicated by non-union requiring repeat bone grafting ($n = 2$) and fracture/infection requiring a staged microvascular fibula arthrodesis.

The rates and types of complications experienced by both the osteoarticular allograft and arthrodesis patients in this study are similar to those reported by other authors. Gebhardt *et al.*[2] reported four complications in three of seven patients undergoing shoulder arthrodesis. These included a wound slough, median nerve palsy and two patients with prominent hardware. Layton *et al.*[15] reported on nine patients treated by shoulder arthrodesis. Two patients died of metastases prior to healing of the arthrodesis and there were complications consisting of infection and fracture in four of the remaining seven patients.[15] Gebhardt *et al.*[3] also reported on twenty patients who underwent osteoarticular allograft of the proximal humerus. In this group, one had a non-union, four fractured and three became infected.[3] O'Connor *et al.*[4] reported osseous union in all eight patients treated with osteoarticular allograft, but four of these patients suffered collapse and fracture of the subchondral region of the allograft. A further patient suffered a fracture and required revision to a second osteoarticular allograft. In the same study, ten patients treated by arthrodesis developed one infection and two stress fractures.[4]

In the present study, the arthrodesis patients tended to have higher scores on all functional measures although only the MSTS 1993 demonstrated a

**Table 2.** *Summary of responses by subjects undergoing shoulder fusion following complications for osteochrondral allograft*

| Case | 1. When you were the most functional after either the osteochrondral allograft or the fusion, after which surgery would you say that your function was better (with respect to ADLs, leisure activities, work)? | 2. Are there any activities that you were able to do after one of the surgeries but not the other (not including any restrictions that the doctor gave you)? | 3. Aside from any complications (fracture, subluxation, infection), how did the level of pain compare after each of the surgeries? | 4. If you had the option of having only one of the two sugeries again, which would you choose? | 5. Why? |
|---|---|---|---|---|---|
| 1 | • fusion, because of increased ROM (70% compared to 45% of shoulder flexion) | • able to golf, swim, waterski after fusion<br>• not working, looking after 1 1/2 year old child | • the same up until the complications | • fusion | • because it is more stable, and I am able to do more activities |
| 2 | • fusion, because my arm feels more like my own (more stable) and not an extra appendage, although I have about the same movement | • can do all the same things (read, walk), doesn't do any sports (never has)<br>• works at desk job, uses arm rest for arm | • much better after the fusion, had severe pain after the allograft (even before she had the problems with subluxation) | • fusion | • because my arm is more stable, and feels more like my own |
| 3 | • osteochondral allograft, because I had better movement | • was able to eat better after the osteochondral allograft, because of better movement, I could bring my hand to my mouth better (although my movement is improving, and I am getting better at it)<br>• other activities about the same (now plays hockey (non-contact), races cars, rides bike, has own business in lawn care) | • about the same (hard to remember) | • allograft—if it was in right arm (dominant arm)<br>• fusion—in the left arm (which he had) | • because the movement was better<br>• because it still functions O.K. |
| 4 | • difficult to tell because of all the complications with the allograft fracture and infection), but it was about the same after both, although my arm feels as though it is improving. | • the same(slight increased ROM after the allograft) although ROM the same in forward flexion (80%) after both | • minimal pain after both surgeries | • fusion | • because the slight increased ROM didn't outweigh the complications, but if I didn't have those, then likely the allograft |
| 5 | • function is about the same although the circulation to the arm seems to be better after the fusion | • the same | • worse after osteochondral allograft, even before the complications happened | • fusion | • because the pain is better |

statistically significant difference between the two clinical groups. The MSTS 1987 and the TESS showed only a trend toward improved function in the arthrodesis group. The lack of statistical significance in these two functional measures must be considered in view of the small numbers of patients in each treatment group as well as the differences in the measures. The MSTS 1987 looks at items that are likely to have low scores in both groups (i.e. range of motion, strength, deformity). Four of the six items of the MSTS 1993 are more likely to vary amongst the two groups. These are pain, function, emotional acceptance and lifting ability, which were generally higher in the arthrodesis patients than the osteochondral allograft patients. There was less of a difference in hand positioning and dexterity. With regards to the TESS scores, most of the patients in both groups were clustered around the mid to high range. This means that all of the patients were able to do basic activities of daily living (eating, bathing, grooming) but experienced difficulty performing high-level activities such as sports, work, endurance and leisure activities. Most of these patients also classified themselves as 'somewhat disabled'.

Although the number of patients in the study was relatively small and five of the patients were in both groups, there is a trend towards improved function after an arthrodesis compared to an osteochondral allograft. Subjective comparison also favoured the arthrodesis. These results are similar to those of O'Connor in which patients treated with primary arthrodesis had higher MSTS 1993 scores than those reconstructed with osteoarticular allografts.[4]

The arthrodesis provides a stable shoulder girdle with motion of the scapulothoracic joint to position the arm in space.[2] Subjective responses from patients in this study indicated a feeling of increased stability and decreased pain after an arthrodesis compared to an osteochondral allograft. These qualitative responses were obtained from patients who had undergone both procedures due to complications from an osteoarticular allograft and were based on patients remembering their 'best' function after their osteoarticular allograft. These results must be interpreted cautiously and in recognition of all the biases imposed in the method.

It is recognized that this study did not include patients with a prosthesis or allograft–prosthesis composite as these reconstructive techniques have not been utilized around the shoulder by our group. Recent work by O'Connor *et al.*[4], however, suggests that the complications and function in patients reconstructed with a prosthesis or allograft–prosthesis composite provides inferior results when compared to an osteochondral allograft or arthrodesis.

In conclusion, complications seemed to be more apparent in patients undergoing osteoarticular allograft. Patients treated with shoulder arthrodesis tended to have better functional scores.

### References

1 Frassica FJ, Sim FH, Chao EY. Primary malignant bone tumours of the shoulder girdle: Surgical technique of resection and reconstruction. *Am Surg* 1987; 53(5):264–9.

2 Gebhardt MC, McGuire MH, Mankin HJ. Resection and allograft arthrodesis for malignant bone tumours of the extremity. In: Enneking WF, ed. *Limb salvage in musculoskeletal oncology*. New York: Churchill Livingston, 1987:567–82.

3 Gebhardt MC, Roth YF, and Mankin HJ. Osteoarticular allografts for reconstruction in the proximal part of the humerus after excision of a musculoskeletal tumour. *J Bone Joint Surg [Am]* 1990; 72-A:334–45.

4 O'Connor MI, Sim FH, Chao EYS. Limb salvage for neoplasms of the shoulder girdle. *J Bone Joint Surg [Am]* 1996; 78-A:1872–88.

5 Malawer MM, Sugarbaker PH, Lampert M, *et al.* The Tikhoff–Linberg procedure: report of ten patients and presentation of a modified technique for tumours of the proximal humerus. *Surgery* 1985; 97(5):518–28.

6 Malawer MM. Tumours of the shoulder girdle. Technique of resection and description of a surgical classification. *Clin Orthop* 1991; 22(1):7–35.

7 Enneking WF, Dunham W, Gebhardt M, Malawer M, Pritchard D. A system for the classification of skeletal resections. *Chir Og Mov* 1990; 75(Suppl. 1):217–40.

8 Friedlaender GE, Mankin HJ, Sell KW. *Osteochondral allografts. Biology, banking and clinical applications.* Boston: Little Brown and Company, 1983.

9 Hawkins RJ, Neer CS. A functional analysis of shoulder fusions. *Clin Orthop* 1987; 223:65–76.

10 Richards RR, Sherman RM, Hudson AR, Waddell JP. Shoulder arthrodesis using a pelvic-reconstruction plate. A report of eleven cases. *J Bone Joint Surg [Am]* 1988; 70-A:416–21.

11 Enneking WF. Modification of the system for functional evaluation in the surgical management of musculoskeletal tumours. In: Enneking WF ed. *Limb salvage in musculoskeletal oncology*. New York: Churchill Livingston, 1987:626–39.

12 Enneking WF, Dunham W, Gebhardt MC, *et al.* A system for the functional evaluation of reconstructive procedures after surgical treatment of tumours of the musculoskeletal system. *Clin Orthop* 1993; 286:24–246.

13 Davis AM, Wright JG, Williams JE, *et al.* Development of a measure of physical function for patients with bone and soft tissue sarcoma. *Quality of Life Research* 1996; 5:508–16.

14 Enneking WF, Spanier SS, Goodman MA. A system for the surgical staging of musculoskeletal sarcoma. *Clin Orthop* 1980; 153:106–20.

15 Layton T, Scarborough M, Vander Griend, RA. Allograft arthrodesis of the shoulder following resection of the proximal humerus. *Contemporary Orthopaedics* 1994; 29(6):421–26.
